# Editorial: Innovations in pharmacogenomics: embracing diversity and clinical application

**DOI:** 10.3389/fphar.2026.1792867

**Published:** 2026-02-13

**Authors:** Maaike Van der Lee, Sarah Cargnin, Elena Peruzzi, Claudia Pisanu, Rossana Roncato

**Affiliations:** 1 Department of Clinical Pharmacy and Toxicology, Leiden University Medical Center, Leiden, Netherlands; 2 Department of Health Sciences, Università del Piemonte Orientale (UPO), Novara, Italy; 3 Department Experimental and Clinical Pharmacology, Centro di Riferimento Oncologico di Aviano (CRO) IRCCS, Aviano, Italy; 4 Department of Biomedical Sciences, Section of Neuroscience and Clinical Pharmacology, University of Cagliari, Cagliari, Italy; 5 Department of Medicine, University of Udine, Udine, Italy

**Keywords:** clinical implementation, diversity, genotyping, pharmacogenes, pharmacogenetics

Pharmacogenomics (PGx) is an enabling component of precision medicine, aiming to improve treatment selection and dosing while reducing adverse drug reactions through the use of genetic information in clinical care. Although genotyping has become more accessible, PGx implementation remains uneven across healthcare systems. Major barriers include limited real-world evidence, incomplete integration into clinical workflows, variability in provider education and in the availability of standardized tools that guide PGx-based clinical decision. An additional and critical challenge is the limited diversity of populations represented in PGx research and reference guidelines, which are still largely based on populations of Caucasian ancestry, leading to the persistent underrepresentation of individuals from other ethnic groups. Beyond issues of ethical equity, this lack of diversity limits the generalizability and clinical validity of PGx-guided interventions due to population-specific allele frequencies, differences in linkage disequilibrium patterns, incomplete variant annotation, and reduced accuracy in genotype-to-phenotype translation across diverse ethnic and genetic backgrounds.

The Research Topic *“Innovations in PGx: embracing diversity and clinical application”* was launched to highlight studies that address these challenges by focusing on clinical translation, implementation in routine settings, ancestry-aware approaches, and emerging biomarkers or analytical strategies. The eleven articles included in this Research Topic reflect the breadth of contemporary PGx, spanning commonly prescribed medicines, high-risk clinical contexts, multiethnic cohorts, and expanding molecular determinants of drug response.

Several contributions revisit established pharmacogenes and widely used therapies, emphasizing the importance of consolidating and clarifying evidence for clinical use. Ibrahim et al. review the relationship between *CYP2C19* polymorphisms and adverse drug reactions associated with proton pump inhibitors (PPIs). While PPIs are frequently prescribed and generally well tolerated, concerns persist regarding long-term adverse effects. The review highlights heterogeneity across observational studies and notes that existing findings are often conflicting or modest, underscoring the need for appropriately designed studies that can better address confounding and long-term outcomes.

Clinical translation is more directly addressed by Mohammed et al. who conducted a systematic review and meta-analysis evaluating point-of-care *CYP2C19* genotyping to guide P2Y12 inhibitor selection in patients undergoing percutaneous coronary intervention. Across randomized controlled trials, genotype-guided antiplatelet therapy was associated with reduced recurrent myocardial infarction and a lower risk of composite major adverse cardiovascular events. These findings support the role of rapid genotyping in time-sensitive settings where treatment decisions must be made promptly.

Implementation science and service design are represented by Alghamdi et al. who describe the integration of a multidisciplinary PGx service into an underserved integrated behavioral health clinic in the United States. Surveyed clinicians reported high perceived potential benefit alongside concerns related to cost, clinical utility, and workflow disruption, and expressed interest in PGx-focused training. The described pharmacist-driven model and the use of preemptive and reactive testing pathways offer a pragmatic example of how PGx services may be integrated into routine care in settings where resources and access constraints are prominent.

In specialized clinical settings, evidence synthesis remains essential to identify actionable signals and define research gaps. The systematic review by Da Costa-Junior et al. On *CYP3A5*, *CYP3A4*, and *ABCB1* polymorphisms in hematopoietic cell transplantation recipients summarizes observational data on tacrolimus and cyclosporine outcomes. The review reports that the presence of the CYP3A5*3 allele influences tacrolimus exposure and related measures, while the evidence for *CYP3A4* and *ABCB1* is more limited.

Besides clinical implementation challenges, a core focus of this Research Topic is genetic diversity and the clinical implications of population variation. Biswas et al. estimate the prevalence of the risk alleles *CYP2C9* (*2, *3) and *VKORC1* c.-1639G>A across global populations using 1000 Genomes data, and map these to PGx-guideline-relevant phenotypes. The analyses show substantial differences across ancestry groups, illustrating how a “one-size-fits-all” approach to dosing may disproportionately affect specific populations.

The interface between pharmacogenetics, drug safety, and regulation is examined by Santos Fidelis et al. in a systematic review of genetic variants associated with metamizole-induced agranulocytosis. The authors summarize the limited available evidence across a small number of studies, with reported signals including HLA-C*04:01 and multiple variants on chromosome nine. By integrating allele frequency data from diverse ancestry groups and a global review of regulatory status, the study highlights the current constraints of the evidence base and argues for larger, well-characterized investigations before genetic findings can reasonably inform regulatory differences across settings.

Real-world evidence from a multiethnic cohort is provided by Alqasrawi et al. who evaluated pharmacogenomic predictors of rosuvastatin discontinuation in the United Arab Emirates. In this prospective observational study, the *ABCG2* rs2231142 variant was associated with an increased discontinuation risk, whereas *SLCO1B1* rs4149056 was not associated with discontinuation in this setting. By linking genetic variability to treatment persistence and LDL-cholesterol change in routine care, this work illustrates how PGx can inform tolerability and adherence considerations in diverse populations.

Acosta-Monterrosa et al. Extend the diversity lens by integrating PharmGKB variant–drug annotations with local allele-frequency data to generate an ancestry-resolved PGx landscape for Colombia. The study quantifies substantial inter-population differences. In particular between African-leaning and European-leaning groups, thereby highlighting the imbalance of global PGx evidence toward European ancestry. Beyond documenting disparities, the authors provide an analytic framework and catalog that can support locally informed prioritization of variants and calibration of precision pharmacotherapy approaches in admixed populations.

The clinical utility of PGx in pediatrics is addressed by Maggo et al. who extracted PGx-relevant information from clinical exome sequencing in a racially diverse cohort of children. The majority of patients carried at least one actionable PGx phenotype across evaluated pharmacogenes. Phenotype frequencies clearly varied based on patients ancestry, highlighting the importance of ancestry awareness.

This topic also includes work pointing to emerging biomarkers and mechanistic hypotheses beyond classical pharmacogenes. Abou Warda et al. Evaluated associations between *SLC5A2* variation and outcomes in heart failure cohorts, including a dapagliflozin-treated population. The observed genotype–outcome patterns differed by treatment status, suggesting that *SLC5A2* variants may be informative for understanding heterogeneity in clinical trajectories and treatment response, while warranting replication and mechanistic follow-up.

Finally, Xu et al. review progress on N6-methyladenosine (m6A) RNA modifications and drug resistance in gastrointestinal tumors, summarizing how “writers,” “erasers,” and “readers” may influence RNA processing and downstream pathways relevant to chemotherapy, targeted agents, and immunotherapy. They explore the biological function and resistance mechanisms of m6A to synthesize new ideas and targets for future treatment.

In summary, the articles in this Research Topic underscore important developments in pharmacogenomics: generating clinically useful evidence in real-world contexts and ensuring that PGx knowledge is applicable across diverse populations. Collectively, they support continued efforts toward inclusive study designs, implementation-ready pathways, and the integration of emerging biomarkers to advance precision medicine.

